# The oral health impact profile and well-being on mothers and preschool children

**DOI:** 10.1186/s12903-024-04137-5

**Published:** 2024-03-22

**Authors:** Bianca Núbia Souza Silva, Lucas Arrais de Campos, João Maroco, Juliana Alvares Duarte Bonini Campos

**Affiliations:** 1 Department of Morphology and children´s clinics, School of Dentistry, São Paulo State University (UNESP), Araraquara, São Paulo, Brazil; 2 Faculty of Medicine and Health Technology, Tampere University, Fin-Medi 1, Arvo Ylpön katu 6, Tampere, FI-33521 Finland; 3 Department of Ear and Oral Diseases, Tampere University Hospital, Tampere, Finland; 4 Faculty of Health Sciences, Institute of Dentistry, University of Eastern Finland, Kuopio, Finland; 5 William James Center for Research (WJCR), University Institute of Psychological, Social, and Life Sciences (ISPA), Lisbon, Portugal; 6 FLU Pedagogy, Nord University, Bodø, Norway; 7 School of Pharmaceutical Sciences, São Paulo State University (Unesp), Araraquara, Brazil

**Keywords:** Oral health, Parent-child, Disease impact profile, Well-being

## Abstract

**Objectives:**

to verify the contribution of mothers’ oral health impact profile to their children’s oral health profile and the contribution of mothers’ well-being and the caries index (dmft) to children’s well-being.

**Methods:**

This is a cross-sectional observational study. Mothers and pre-school children enrolled in public schools in the municipality of Araraquara-SP took part. The Oral Health Impact Profile Questionnaire (OHIP-14), Satisfaction with Life Scale (SWLS), Early Childhood Oral Health Impact Scale (ECOHIS) and the Autoquestionnaire Qualité de Vie Enfant Imagé (AUQEI) were used. To diagnose caries, a clinical examination was carried out to calculate the dmft index. Path analysis was carried out and the path coefficients were estimated (β) and evaluated using the z-test (α = 5%).

**Results:**

443 children took part (5.19 ± 0.64 years; 52.4% boys) with an mean dmft of 1.31 ± 2.19. The mean age of the mothers was 33.4 ± 7.01 years. There was a significant impact of dmft and maternal well-being on the child’s subjective well-being (s^2^explained = 43%). The mothers’ oral health impact profile and the child’s caries experience had a significant influence on both the child (OHIP: β = 0.22; *p* < 0.001; dmft: β = 0.48; *p* < 0.001) and the family (OHIP: β = 0.29; *p* < 0.001; dmft: β = 0.32; *p* < 0.001). The child’s dmft (β=-0.10; *p* = 0.005) and the mothers’ subjective well-being (β=-0.61; *p* < 0.001) had a significant impact on the child’s subjective well-being.

**Conclusion:**

The mothers’ oral health impact profile and the child’s caries experience had an impact on both the child and the family. Mothers’ subjective well-being and caries experience should be considered when assessing the subjective well-being of Brazilian preschool children.

## Introduction

Oral health is a broad concept and its assessment must consider physiological and psychosocial aspects in addition to the clinical characteristics of the mouth and knowledge of the health/disease process [1]. Oral pathologies, such as caries, have physical, social and psychological consequences that can have a negative impact on the lives of children and their families [2] and, considering their impact on people’s quality of life [3] theoretical models have been proposed [4] to explain this relationship.

One of the most widely used models considers aspects such as the profile of functional limitation, pain or discomfort, disability and injury and their impact on people’s quality of life and well-being [5]. Based on this model, the quality of life related to oral health began to be researched. According to John et al. [6], the concept of quality of life related to oral health involves four dimensions: oral function, orofacial appearance, orofacial pain and psychosocial impact, which reflect how oral problems impact on each person’s life. Thus, considering this theory, it can be concluded that the aim of oral health care should involve not only the treatment of oral problems, but also the improvement and/or restoration of physiological and psychosocial functions [7].

It is important to emphasize that the earlier this type of approach is carried out, the greater the likelihood of educational and preventive intervention aimed at promoting favorable health behaviors and, therefore, childhood presents a strategic phase for intervention [8, 9]. It should also be considered that in the first years of a child’s life, the process of primary socialization takes place, where the individual incorporates habits and values from highly significant people, usually parents, and this process is full of affection [10]. At this stage, not only cognitive but also affective development takes place and children learn their roles and attitudes in the world, including health-related practices [10, 11]. The study of child development in the context of the family is therefore important. Despite the important changes in the profile of the contemporary family in Brazil, women still play a key role in the process of primary socialization, especially with regard to psychological and emotional aspects, and the mother therefore plays a fundamental role in the child’s development [11, 12]. It may be suggested that there is a close relationship between the habits and attitudes of the mother and the child, since they are still the main caregivers and have more parental interaction with the child [11]. Given this fact, we hypothesize that there may be a significant relationship between the oral health impact profile of the mother and the child and between the mother’s well-being and the child’s well-being, and based on this premise, the identification of these aspects may open up space for the incorporation and/or modification of habits in favor of the development of positive health attitudes by both mothers and children.

To assess the oral health impact profile of adults, one of the most widely used instruments in the literature is the *Oral Health Impact Profile* (OHIP). The OHIP, was originally proposed by Slade and Spencer [13] in English and its full version consists of 49 items distributed in seven factors (functional limitation, physical pain, psychological discomfort, physical disability, psychological disability, social disability and handicap). The reduced version of the OHIP consists of 14 items (OHIP-14) distributed in the same seven factors [14] however, it is also possible to find suggestions for second and third order hierarchical models [15] and also unifactorial [16] of this instrument. For children, the Early Childhood Oral Health Impact Scale (ECOHIS) [17] was developed, consisting of 13 items distributed into two factors (child subscale and family subscale) which seek, from the point of view of parents and/or guardians, to identify the impact of the child’s oral health on the child’s life and that of their family. Another aspect to be highlighted is that both ECOHIS and OHIP are psychometric scales developed to estimate the impact of oral problems on the lives of individuals, which only deals with one aspect that may or may not impact on well-being or quality of life and, therefore, this concept cannot be confused or treated as quality of life itself [6].

In addition to identifying the impact of oral health on the lives of mothers and children, it may also be relevant to check how it affects their well-being. Subjective well-being is a complex concept, made up of an affective component involving experiences related to emotions and a cognitive component inherent in life satisfaction, which refers to an individual’s assessment of their own life, which can be made in relation to life as a whole or considering specific components [18]. Life satisfaction is a self-report measure and its evaluation involves a comparison between situations experienced by the individual and the standards they have internalized [19]. Considering that children internalize the roles and attitudes of their family members, making them their own and externalizing their own being in the social world [10, 11], research into the impact of parents’ life satisfaction on children’s well-being may be relevant.

Among the instruments proposed to assess subjective well-being in adults is the *Satisfaction With Life Scale* (SWLS), that was developed by Diener [20], It is a unifactorial scale made up of five items and has been widely used in the literature. For use in children, we have the *Autoquestionnaire Qualité de Vie Enfant Imagé* (AUQEI) proposed by Manificat and Dazord [21], composed of twenty-six items, divided into four factors (autonomy, leisure, functions and family). In 2023, Silva et al. [22] presented the Portuguese version of the instrument and verified adequate psychometric indicators for a sample of Brazilian pre-school children.

In view of the above, this study was carried out with the aim of verifying the contribution of the oral health impact profile in the lives of mothers to the oral health profile of their children and the contribution of the children’s caries index and the well-being of mothers to the well-being of children.

## Methods

### Procedures and ethical aspects

This study was carried out in the Children’s Education and Recreation Centers (CER) in the municipality of Araraquara - São Paulo, and permission was obtained from the Municipal Department of Education. To collect the data, the lead researcher scheduled a visit to each CER to present the study and obtain agreement to include the institution. Araraquara is a Brazilian municipality located in the central region of the state São Paulo with an estimated population of 242.228 corresponding to a population density of 241.35 inhabitants/km^2^. It should be noted that Araraquara was chosen for convenience and access by the researchers. During this visit, appointments were made for the questionnaires to be filled in by the mothers and for clinical oral examinations to be carried out on the children. The STROBE (Strengthening the Reporting of Observational studies in Epidemiology) [23] tool was used to help design the study and report the results.

### Study design and sample design

This is a cross-sectional observational study. Children aged between 4 and 6 (pre-school) enrolled in municipal public schools in Araraquara-SP and their mothers were invited to take part.

The minimum sample size was calculated using α = 5%, β = 20%, ε = 12.5%, *N* = 2.272 (total number of pre-school children enrolled in the CERs in Araraquara) and the estimated prevalence of caries in 5-year-old children in the state of São Paulo estimated by the Projeto Saúde Bucal Brasil (Brazil Oral Health Project) [24] (*p* = 41.8%). Thus, the minimum sample size estimated was 298. Considering the possibility of a loss rate of approximately 15% of the data, the sample size was corrected and estimated at 351.

To characterize the sample, information was collected on the child’s sex, age and caries experience (dmft index), age (years), parents’ economic level and schooling, and the presence/absence of work among mothers. The economic level of family members was estimated based on purchasing power using the Brazilian Economic Classification Criterion – ABEP [25].

### Oral clinical examination

Dental caries was diagnosed based on the World Health Organization (WHO) [26] criteria using the dmft (number of decayed, missing due to caries and filled teeth in the primary dentition). The oral exam was carried out in a school environment, under natural light, by a single examiner previously calibrated in a pilot study. A total of 25 children took part in the pilot study. The dmft index was assessed twice with a one-week interval between tests and intra-examiner reproducibility was excellent (Intraclass Correlation Coefficient = 0.998; 95%CI= [0.995–0.999]).

### Measuring instruments

The mothers’ oral health impact profile was estimated using the reduced Portuguese version of the Oral Health Impact Profile (OHIP-14) [27]. This instrument consists of 14 items divided into 7 first-order factors (Functional Limitation, Physical Pain, Psychological Discomfort, Physical Disability, Psychological Disability, Social Disability, Disabilities). The response scale for the items is a 5-point Likert scale (0: never to 4: always). The theoretical proposal for operationalizing the concept used in this study was a unifactorial model which deals with one of the possible theoretical models of the OHIP-14 and which was previously tested by Campos et al. [16].

For the children, the Portuguese version of the *Early Childhood Oral Health Impact Profile* proposed by Tesch et al. [17]. The ECOHIS consists of thirteen items, of which nine (items 1 to 9) assess the impact of oral problems on the child (child subscale) and four (items 10 to 13) assess the impact of the child’s oral problems on their family (family subscale). The items were answered on a 5-point Likert scale (1: never to 5: very often). All the items were answered by the child’s mother.

Mothers’ subjective well-being was estimated using the Satisfaction with Life Scale (SWLS) originally proposed in English by Diener [20] with a unifactorial model and made up of 5 items with 7-point Likert-type answers (1: Strongly disagree to 7: Strongly agree). This study used the Portuguese version of the instrument proposed by Gouveia et al. [28]. For the children, the Portuguese version of the *Autoquestionnaire Qualité de Vie Enfant Imagé* (AUQEI) proposed by Assumpção Júnior et al. [29]. The instrument consists of 26 items and is answered using a scale of five images of faces with different emotional states ranging from very unhappy to very happy. The factor structure of the AUQEI used was unifactorial, the same as previously presented and tested by Silva et al. [22].

### Validity and reliability

To check the fit of the theoretical models proposed for each instrument to the study samples (mothers and children), confirmatory factor analysis (CFA) was performed using the *Weighed Least Squares Mean and Variance Adjusted* (WLSMV). The goodness of the fit was with the *Comparative Fit Index* (CFI), the *Tucker-Lewis Index* (TLI), the Root *Mean Square Error of Approximation* (RMSEA) and the *Standardized Root Mean Square Residual* (SRMR). The fit was considered acceptable when CFI e TLI ≥ 0.90; RMSEA < 0.10 and SRMR < 0.08 ^30^. The fator loading (λ) was considered adequate when ≥ 0.50. Convergent validity was assessed using the average variance extracted (AVE) and was considered adequate if ≥ 0.50 ^30, 31^. Reliability was estimated using the ordinal alpha coefficient (α) and omega (ω) and values α and ω ≥ 0.7 were indicative of satisfactory internal consistency [30, 31]. The analyses were carried out using the “lavaan” [32] and “semTools” [33] packages in the R program [34]. To adjust the OHIP model to the sample, item 14 was excluded because it violated the normality assumptions (absolute values of asymmetry = 3.65 and kurtosis = 14.01). After this refinement, all the instrument models showed an adequate fit to the study samples (mothers and children), pointing to the adequate validity and reliability of the data presented here (Table 1).

**Table 1:** Psychometric indicators obtained when adjusting the factor models of the different instruments to the study samples (mothers and children; *n* = 443)

	Instruments			
Indicators	OHIP-14	ECOHIS	SWLS	AUQEI
Peso fatorial (λ)	0.72–0.90	0.65–0.88	0.71–0.90	0.71–0.92
Comparative Fit Index (CFI)	0.98	0.95	0.99	0.98
Tucker-Lewis Index (TLI)	0.98	0.94	0.99	0.98
Root Mean Square Error of Approximation (RMSEA)	0.10	0.09	0.10	0.08
Standardized root mean square residual (SRMR)	0.05	0.07	0.02	0.05
Average Variance Extracted (AVE)	0.70	0.62–0.66	0.65	0.70
Ordinal alpha Coefficient (α)	0.96	0.86–0.93	0.90	0.98
Ômega Coefficient (ω)	0.95	0.80	0.87	0.93

### Statistical analysis

Initially, the mean factor scores of each measurement instrument (OHIP-14, ECOHIS, AUQEI) were calculated and a path analysis was conducted to estimate the contribution of the mothers’ oral health impact profile to the oral health profile of their children and to also verify the contribution of the children’s caries experience and the mothers’ well-being to the children’s well-being. The mean scores of the instrument factors were compared between the group of children who had some caries experience (dmft > 0) and those with dmft = 0 using Student’s t-test (α = 5%). This comparison was possible after strong measurement invariance was confirmed (M0: configural, M1: metric, M2: scalar, M3: strict models; scalar and strict invariance were attested when ΔCFI_M2−M1_ and ΔCFI_M3−M2_ <|0.01|) [35] of the models of the different instruments between the two groups.

The standardized coefficients (βpadronizado) used to indicate the relationships between the variables were estimated using the maximum likelihood method and their significance was assessed using the z-test (α = 5%). Caries experience was defined based on the index dmft considering two categories (0: dmft = 0; 1: dmft > 0) and for sex, a score of 0 was adopted for males and 1 for females. The final model consisted only of statistically significant trajectories (*p* < 0.05). IBM SPSS and AMOS (v. 28.0 IBM Corp., Armonk, NY, USA) were used.

## Results

A total of 443 children participated in the study (age: mean = 5.19; standard deviation (SD) = 0.64 years; 52.4% male). The mean age of the participants’ mothers was 33.4 (SD = 7.01) years. The majority of these mothers reported being married (*n* = 276; 63.6%; single: *n* = 124; 28.6%, separated: *n* = 30; 6.9%, widowed: *n* = 4, 0.9%), working (*n* = 296; 67.6%) and belonging to economic strata B (estimated mean family income R$ 7,053.00; *n* = 200; 45.1%) and C (estimated mean family income R$ 2,165.00; *n* = 205; 46.3%). The mean dmft of the children was 1.31 (SD = 2.19) and 41.5% of the children had some caries experience (dmft > 0). The descriptive statistics of the scores obtained for each factor of the instruments according to caries experience are shown in Table 2. There was a greater impact of oral health on mothers and children when they had caries experience, which was expected. With regard to subjective well-being, the mothers of children with caries experience had lower mean scores for subjective well-being.

**Table 2:** Descriptive statistics of the instruments according to caries experience

Components	No caries exprience (*n* = 259)	With caries experience (*n* = 184)	Total (*n* = 443)	T(p)
OHIP	0.59 ± 0.81	0.91 ± 0.92	0.72 ± 0.87	-3.82(< 0.001)
ECOHIS (Children)	1.12 ± 0.23	1.67 ± 0.62	1.35 ± 0.51	-11.32(< 0.001)
ECOHIS (Family)	1.15 ± 0.43	1.61 ± 0.72	1.34 ± 0.61	-7.69(< 0.001)
SWLS	5.06 ± 1.36	4.12 ± 1.49	4.67 ± 1.48	6.86(< 0.001)
AUQEI	2.98 ± 0.67	2.55 ± 0.69	2.80 ± 0.71	6.50(< 0.001)

**Fig. 1:**
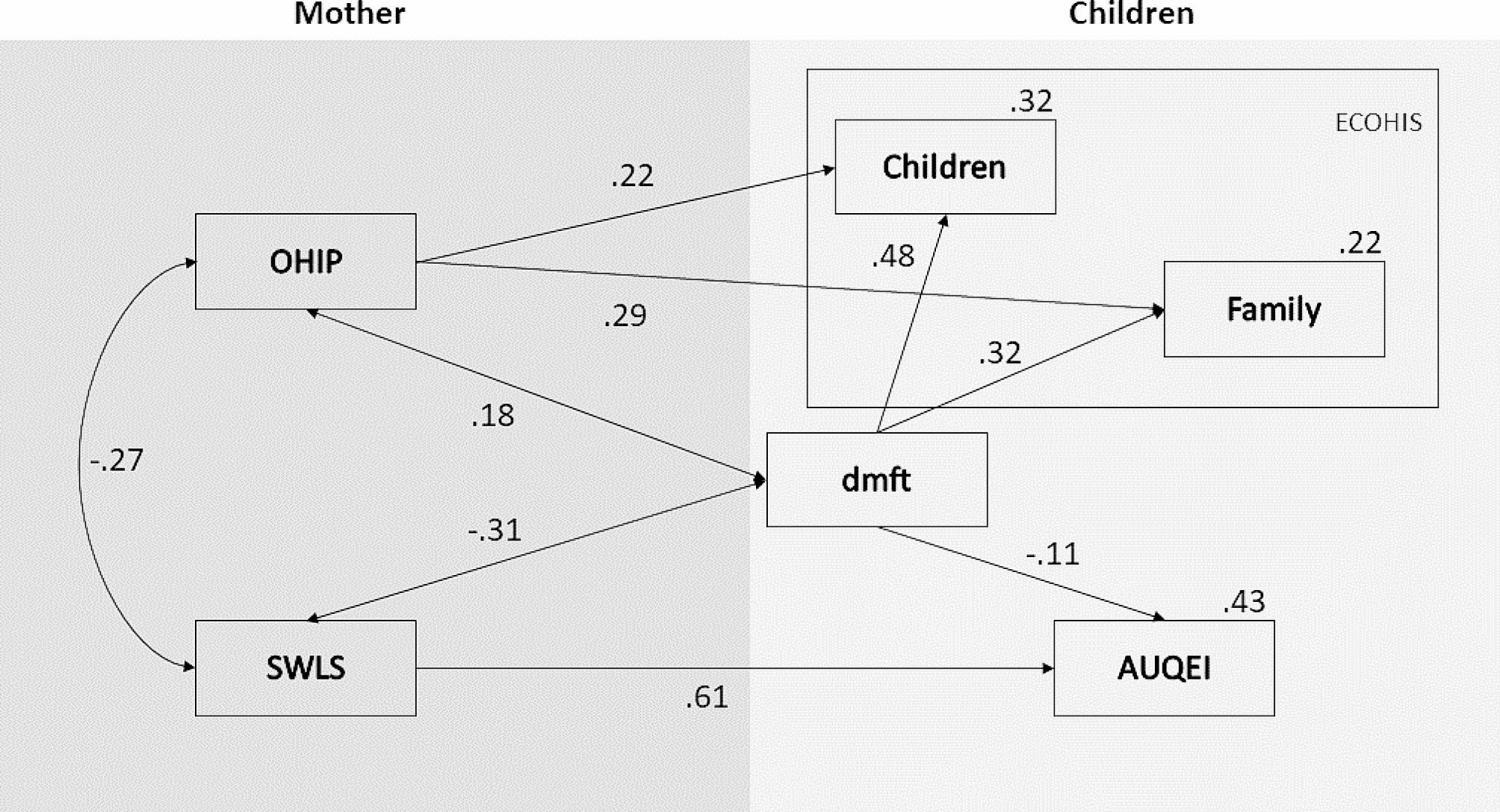
Path analysis was carried out to verify the impact of the caries index (dmft) and mothers’ subjective well-being (SWLS) on children’s well-being (AUQEI), as well as dmft and the impact of oral health on mothers’ lives (OHIP) on the impact of oral health perceived by the child and the family

Sex had no significant impact on the child’s subjective well-being and was therefore removed from the final model. Figure 1 shows the final model composed only of the statistically significant trajectories (*p* < 0.05). There was a significant impact of dmft and maternal well-being on child well-being, together explaining 43% of the variability in child well-being (Table 2). In addition, the mother’s oral health impact profile and the child’s caries experience explained 32% of the variation in the impact of oral health on the child’s life as perceived by the mother and 22% on the family’s life (Fig. 1).

## Discussion

The results presented in this study show a significant contribution of the oral health impact profile presented by the mothers and the child’s caries experience to the oral health impact profile in the lives of both the child and the family. A child’s health is significantly affected by parental factors, the family plays a significant role in establishing practices for health maintenance and care [11, 12].

Our results are in line with the findings presented in the literature [11, 12], indicating a significant association between the oral health of mothers and the oral health of their children. This result may be related to the fact that children, especially during early childhood, copy their parents’ behavior, since it is during this phase of primary socialization that they spend most of their time with their mothers, who are the main caregivers and play a direct role in their children’s health behavior [10, 11]. This parent-child relationship has been analyzed and studies show that children’s positive oral health behaviors are related to the behaviors displayed by their parents [12, 36]. Buldur and Oguz [36, 37] showed a direct relationship between the oral health behavior of children and their parents, concluding that if parents have better oral health habits, their children will also have better oral health habits. A showed a relationship between the brushing habits of mothers and children. It is therefore important to emphasize that a family-based approach can be effective in preventing caries disease.

It is important to note that the choice to have mothers fill out the ECOHIS rather than another person responsible, such as fathers, was based on the standardization of the data, as well as the arguments put forward by Borsa and Nunes [38], who state that despite the changes in social roles that are currently taking place in family configurations, childcare is still the primary responsibility of women, with a greater tendency to be involved in child rearing. The choice of age (pre-schoolers) for the study was also based on the literature [11, 39, 40], which points out that young children have their mothers as the building blocks of reality and, therefore, the family environment is strictly related to the child’s physical, psychological and social development and health promotion practices are also learned in this system.

In addition to impacting the family, caries causes discomfort, swelling, pain, loss of sleep, changes to the child’s diet, such as difficulty chewing and swallowing, as well as embarrassment at the shape or absence of teeth [2, 41]. In line with our results, Pakkehsal et al. [2] found a significant relationship between caries disease in the lives of 350 children aged between 3 and 6 and their parents. In addition, a systematic review and meta-analysis concluded that the disease has a negative impact on the lives of preschool children who have caries when compared to children who do not. This can be justified by the negative consequences on the physical and functional aspects of children’s lives caused by the disease [2, 41, 42]. It is important to note that the study of chronic diseases is essential due to their impact on the health-related quality of life [15] and well-being of individuals [43].

Another relevant contribution of this study is the presentation of evidence related to the impact that the well-being of mothers has on the well-being of children, which opens the way for a more robust discussion regarding the importance of planning health actions that include mothers and that address not only issues related to oral health, but also broader aspects such as well-being and lifestyle. Studies corroborate our findings [40, 43] and document the strong relationship between mothers’ well-being and positive aspects of their children’s development.

The literature [40, 43] reports that when mothers go through moments of negative experiences, which can lead to emotional changes, these emotions are transmitted to the children during the interaction, which can result in less affection being exchanged in the relationship, fewer smiles and encouragement, and the presence of more criticism and reprimands, thus affecting the child’s well-being. This relationship is therefore bidirectional and interdependent: mothers and children react directly and indirectly to each other’s behavior and continually adjust as a way of reaffirming values and beliefs, modulating their way of acting [44].

Given this, the well-being and emotional regulation of parents is important, as it has an impact on the well-being of children. This fact reinforces the need for interventions aimed at the family, and not exclusively at children. To this end, education and prevention programs, public policies and health professionals need to consider this relationship and invest in actions aimed at the physical, mental and social well-being of families [45], especially mothers, since maternal mental health can be a predictor of the mental health and well-being of children [43]. These findings reinforce the strong bond between mothers and their children, supporting the importance of the maternal connection as being relevant to children’s development.

A limitation of this study is its cross-sectional design, which makes it impossible to establish cause and effect relationships. Despite this limitation, the study provides information on the relationship and impact of mothers and caries disease on the lives of pre-school children, which may be relevant to professionals, researchers and public policy managers for the development of strategies to promote healthy habits based on mothers, since they play an important role in decision-making when it comes to the general health and well-being of their children [12]. .

## Conclusion

The oral health impact profile of the mothers’ lives and the child’s caries experience had an impact on both the child and the family. Mothers’ subjective well-being and caries experience should be considered when assessing the subjective well-being of Brazilian preschool children.

## Data Availability

No datasets were generated or analysed during the current study.
